# Care Partners' Information Needs When Caring for a Family Member With Parkinson's Disease: An Explorative Study

**DOI:** 10.1111/scs.70277

**Published:** 2026-06-05

**Authors:** Helena Larsson, Maria Haak, Peter Hagell, Carina Hellqvist

**Affiliations:** ^1^ Faculty of Health Science Kristianstad University Kristianstad Sweden; ^2^ The Research Platform for Collaboration for Health, Kristianstad University Kristianstad Sweden; ^3^ Department of Health, Medicine and Caring Sciences. Division of Nursing Sciences and Reproductive Health Linköping University Linköping Sweden; ^4^ Department of Neurology Linköping University Hospital Linköping Sweden

**Keywords:** care partners, information, interview, Parkinson's disease, thematic analysis

## Abstract

**Background:**

Parkinson's disease (PD) is a progressive neurodegenerative disorder that increasingly impacts many aspects of everyday life for those affected. The involvement of family members becomes increasingly important as PD progresses. Although family members are often willing and ready to help and support as a care partner, their role can be demanding, with consequences for their own health and well‐being. One way to offer care partners support could involve providing information to increase insight into the progress of PD.

**Aim:**

To explore care partners' information needs when caring for a family member with Parkinson's disease.

**Methods:**

Fifteen care partners were interviewed in focus groups, dyadic or individually, with open‐ended questions. The interviews were recorded and transcribed. Data were analysed using reflexive thematic analysis.

**Results:**

The analysis revealed four themes reflecting care partners' information needs in their caregiving role: (1) to be prepared when symptoms limit daily life; (2) to preserve participation and social life; (3) to find reassurance together with others; and (4) to cope with feelings of uncertainty.

**Conclusions:**

The results demonstrate the need for personalised information meeting the care partners' physical, social, emotional and existential needs. This calls for holistic information‐based interventions built on collaboration between healthcare professionals, the person with PD and the care partner, aimed at promoting care partners' well‐being and helping them support their family member with PD. The themes identified here may be seen as a framework for such information‐based interventions.

## 1 Background

Parkinson's disease (PD) is a chronic neurodegenerative disease that mainly affects older people. Globally, PD has doubled in the last 25 years, and it is estimated that 8.5 million people worldwide are living with the diagnosis [1]. Since available treatment only is symptomatic and cannot slow or cure the progression of PD, persons affected by the disease often live for many years with the diagnosis. While the cause of the disease is not fully understood, it is thought to involve a combination of genetic vulnerability and external factors [2, 3]. PD pathology is mainly described in terms of the loss of dopaminergic neurons leading to movement problems characterised by balance problems, rigidity, tremor and slowed movements. However, PD is systemic and affects several brain functions in addition to the dopaminergic system [2]. Thus, symptoms also include a range of non‐motor symptoms such as cognitive difficulties, communication problems and neuropsychiatric symptoms [3]. Persons with PD (PwPD) have substantial variation in symptoms and rate of progression, making it difficult to know how the disease will affect each individual. Additionally, daily functioning and the effects of medication can vary significantly and unpredictably from day to day and even hour to hour. However, even with advanced disease, most PwPD live in their own homes, largely relying on support from family members to manage everyday life with PD [4, 5].

The concept of a ‘care partner’ highlights an approach whereby a partner/family member/close friend and the person with a condition willingly deal with the disease together as a joint responsibility [6]. This concept aligns well with the situation described by people close to a PwPD [7], who live with uncertainty about the progression of PD [4]. The involvement of the care partner becomes increasingly important as PD progresses, and they are often involved in supporting and helping the PwPD with tasks in everyday life, as well as managing the disease and medical treatment [8, 9]. In later stages of PD, their help and support are what enables PwPD to remain in their own homes [10]. Although family members may be willing and ready to help and support as care partners, the role can be both physically and mentally demanding [11, 12], often with consequences for the care partner's own everyday life, health and well‐being [13], and for the relationship with the PwPD [14]. Although care partners' support is crucial, their experiences of being a carer and their needs are insufficiently considered in clinical practice and research [15].

Care partners have described a lack of support from healthcare services in understanding and managing PD in everyday life [16]. In addition, care partners are often offered no information, even though it is well known that they become increasingly involved in managing everyday life for the PwPD as the disease progresses [11]. Here, *information* refers to both knowledge and what we learn from each other through, for example, social interactions [17]. One way to support the care partners of PwPD could be to increase their knowledge and understanding regarding the disease and its therapy and to provide self‐care advice to increase their well‐being. The first step towards an exploration of what information care partners need in relation to caring for a PwPD is to ask them. Therefore, the aim of this study was to explore care partners' information needs when caring for a family member with PD.

## 2 Method

### 2.1 Design

This study has an explorative design with an inductive approach [18]. Through open‐ended questions, 15 care partners were interviewed in focus groups, dyadic or individually about their needs for information when being a care partner to a PwPD. The interview transcripts were analysed using reflexive thematic analysis (TA) [19, 20]. The Consolidated Criteria for Reporting Qualitative Research (COREQ) were used when presenting the study [21].

### 2.2 Setting

In Sweden, PwPD are supposed to be managed by a specialised Parkinson's team of healthcare professionals [22]. Such PD teams are available at some hospitals in neurological, geriatric and rehabilitation clinics across the country. Whilst care partners are welcome to accompany the PwPD on visits, the focus of the team is on the PwPD.

### 2.3 Procedure

The study was conducted in the middle and south of Sweden. The directors of the neurological clinics at two hospitals with specialised PD teams, as well as one carers association in one municipality, were contacted. After receiving permission from the appropriate leadership, healthcare professionals at the hospitals and the chair of the carers' association distributed a study information letter to care partners who might be eligible to participate in an interview. The inclusion criteria were self‐identification as a care partner to someone diagnosed with PD, the ability to express oneself orally and an understanding of the Swedish language. If care partners showed interest and gave permission, their name and contact details were provided to the researchers, who contacted potential participants and provided oral and written information about the study. Twenty care partners were identified, five of which declined further participation. Those who agreed to participate (*n* = 15) were provided with the date and place for a focus group interview. In addition, a question was asked about the length of time since the PwPD received the PD diagnosis. This was done in an attempt to gather care partners in similar situations in the same focus group. Six care partners were unable to attend or felt uncomfortable attending a group interview and were offered an alternative interview. Two of these care partners took part in a focus group interview together with their PwPD; three took part in individual interviews; and one took part in a dyadic interview. Consequently, three focus group interviews (*n* = 11), three individual interviews (*n* = 3) and one dyadic interview (*n* = 1) were conducted between autumn 2023 and spring 2024. Table 1 provides an overview of the participants.

**TABLE 1:** Overview of the interviews and participant characteristics (*N* = 15).^a^

Type of interview	Number of care partners	Living together/alone	Men/women	Age (years), min–max	Education: lower and upper secondary school/college/university	Participated in/received information/training about PD, no/yes	Relationship with PwPD: child/parent/partner	Time (years) since PD diagnosis, min–max
Focus group	4	4/0	1/3	62–76	1/1/2	3/1	0/0/4	2–19
Focus group	5	2/3	1/4	68–84	0/3/2	4/1	0/2/3	5–35
Focus group^b^	2	2/0	0/2	68–71	0/2/0	2/0	0/0/2	17–20
Dyadic	1	1/0	0/1	86	1/0/0	0/1	0/0/1	14
Individual	3	3/0	1/2	32–72	0/1/2	2/1	2/0/1	4–20
Total	15	12/3	3/12	32–86 (mean, 71)	2/7/6	11/4	2/2/11	2–35 (mean, 13.2)

Abbreviations: PD, Parkinson's disease; PwPD, person with Parkinson's disease.

^a^ All data are *n* unless otherwise stated.

^b^ Together with their family members with PD.

### 2.4 Data Collection

The interviews started with a presentation of the study and of the researchers conducting the interviews. Next, participants were invited to introduce themselves and narrate their relationship and everyday life with a PwPD. After that, the following overarching questions were used to stimulate reflections and discussions [23]: what are your experiences of information gained during the trajectory of PD? What information do you need in relation to being a care partner to a PwPD? When and how do you need information? In addition, probing questions were used to deepen the discussions. For example, how did you experience the situation? What would you have needed in that situation? Following the interviews, demographic information was collected, such as gender and age, by asking all participants to fill in a form with predefined questions (Table 1).

During the focus group interviews [23], one of the researchers (first or last author) acted as a moderator and led the discussion, while the other researcher acted as an observer, taking notes and asking probing questions. The dyadic and individual interviews were conducted by one researcher (first or last author). The focus groups and the individual interviews were conducted at the hospitals (*n* = 5) or at a senior centre in the municipality (*n* = 1). The dyadic interview was conducted in the participants' home according to their wish. The focus groups lasted between 70 and 100 min, the individual interviews lasted between 32 and 57 min, and the dyadic interview lasted 65 min. The interviews were audio‐recorded and transcribed verbatim.

### 2.5 Analysis

The interview transcripts were analysed using reflexive TA [19, 20]. This method does not follow a certain procedure; rather, the researcher goes into the data with a reflective approach, reading, thinking and searching for patterns of meaning in relation to the aim. In line with the description by Braun and Clarke [19], the process included continuous movement between the interview transcripts and our reflections and discussions. The analysis started with the first author reading the transcripts from the focus group interviews, then the interview with the dyad, and finally the individual interviews. Only data involving the care partners' voices were included in the analysis. During the second reading, markings were made in the text to highlight specific pieces of content (meaning units) of interest in relation to the aim. This resulted in 121 meaning units, approximately 5–20 rows long each. In the next step, each meaning unit was provided with a shorter code, such as ‘pain limits everyday life’ or ‘need for security when thinking about the future’. The codes were then sorted based on how they were related, and the sorting was discussed by the first and last author several times. From the sorted codes, preliminary themes were generated, reflected upon and discussed with all authors on two different occasions, resulting in the final themes. Table 2 provides an example of the analysis process.

**TABLE 2:** Examples of meaning units, codes and a theme.

Example of meaning unit	Code	Theme
… and then there's a … a certain brain fatigue … so that he can't really focus for long and … so I can experience that, and that I can say the same thing two—three –four times and still it disappears, I've noticed it more recently, so I'm worried about this Alzheimer's threat that you have, because I have … we've both read a lot, we read and read and find out things, so that it's well … that's probably my biggest worry now, I think, that it's going to go to the brain … As we've always had an ability to talk about everything and discuss everything, good and bad and all that, then it disappears, I think, I get really worried, that's probably my biggest issue, I think, now (Individual interview 12)	Worry about the future, a changed relationship	Coping with uncertainty
I: So he forgets very quickly … and I have to keep track of all the physician's appointments and medication, and I share the medication and manage it, so no, he wouldn't be able to do that now … unfortunately …. II: But then maybe I'm a bit on the other side, that I like to take over a bit too much, I have to slow down sometimes and sort of decide that no, now you do this yourself, I'll come when you're ready, call if you need me, so …. I: It's a balancing act, when you help too much (Focus group interview 15).	Helping too much or too little, a balancing act, difficult to know	
… there are a lot of thoughts … that you want to do as much as you can, when you know that it's like … it can't be cured, but you know … you don't know the pace or anything like that either, so then there's a lot of thinking about it … when is it time for this, or when will this not work, how long will this work … or what kind of direction, because it's also like this; some people are affected in different ways and things like that, so there are obviously a lot of thoughts about it … actually, I think it's about, that it felt unfair, and it's not a fun disease … so, for quality of life and so … and at the end as well … the uncertainty … (Individual interview 13)	Many concerns, unfairness, uncertainty	

### 2.6 Ethical Considerations

The study was approved by the Ethical Review Board of Sweden (2023‐02625‐01). Informed consent was obtained orally and in writing from all participants before data collection began. Participants were informed that participation was voluntary and would not affect the care provided to their PwPD, and that they could withdraw their consent at any time without an explanation for doing so.

## 3 Results

The analysis revealed four themes reflecting care partners' information needs in their caregiving role: (1) to be prepared when symptoms limit daily life; (2) to preserve participation and social life; (3) to find reassurance together with others; and (4) to cope with feelings of uncertainty. Each theme is described using quotations to provide examples from the interviews. When a quotation is from a focus group interview, Roman numerals indicate the contributions from different care partners.

### 3.1 To Be Prepared When Symptoms Limit Daily Life

The care partners asked for repeated information on detecting and managing symptoms at different stages of PD, as the disease can last for many years. They described several symptoms that affected the PwPD, such as tremors, dyskinesias, sleeping difficulties and problems with mobility. Initially, at the time of diagnosis, they only noticed a shaking arm or a dragging leg; however, as PD progressed, the symptoms increased and became more obvious.

As the years went by, the care partners became increasingly aware of symptoms that interfered with the PwPD's daily life, such as a risk of falling, cognitive decline or speech difficulties. To be prepared, when such symptoms started to appear, the care partners wished to receive gradual information about these symptoms and about what other symptoms to expect as the disease progressed. They highlighted that preparedness would allow them to prepare themselves and other family members and friends in order to reduce suffering for the PwPD. However, they did not want too much information early in the disease trajectory. The examples given included wishing they had known beforehand that medications can cause hallucinations or that a person with PD suddenly can forget how to perform a movement, such as how to swim. The care partners noted that it is extremely important to be prepared for such a difficult or potentially dangerous situation to arise because of PD, and they requested information about such situations and how to manage them if they occurred. The care partners described how the responsibility for dealing with difficult situations was placed on them and that they were the ones expected to lead and guide the PwPD to manage symptoms that limit everyday life.

I: We've … had the disease for about 10 years, he got it at the same time as he retired … but he's felt very well, so we haven't worried, since he started taking medication, he's functioned more or less as usual, but gradually less active … and it's worked until … a year and a half ago, when a very sudden deterioration occurred that may not be visible to him, but which I experience, and he experiences poor sleep, pain … in different parts of the body … and then we realised that we've been in a ‘shimmer’ [being in the early stages of PD].

II: We thought that Parkinson's wasn't that bad, but then we understood and started reading and looking for information about what's to come, and that it doesn't get better, but it gets progressively worse (Focus group interview 11).

To feel prepared for the challenges of PD, care partners called for tailored information about diet, sleep and exercise in relation to PD, with specific advice based on the phase of PD. They noted that it was a challenge that the disease manifests in different ways, making it difficult to know how to notice symptoms. The care partners expressed that the symptoms always were on their minds, to be prepared for situations that might arise.

I'm anxious when he wants to carry on building and climbing … and then he gets depressed when his body doesn't do what he wants … so there … I don't know, there are certain things like that, like worrying about falling, and then he fights at night. So, we had to build a pillow between us so that I don't get hit in the face … or he falls out of bed … things like that are in a carer's mind all the time. It would be nice if someone had told you that situations like that might come (Individual interview 12).

### 3.2 To Preserve Participation and Social Life

To preserve participation in social life, care partners described needing information about how to think in a new way and find strategies in relation to social gatherings, instead of avoiding situations that arose in social contexts. They described how their social network decreased as the PD progressed, both for the PwPD and for themselves. It became more problematic to participate in gatherings with family and friends and to be involved in community activities. Maintaining social contacts became a challenge when it was no longer possible for the PwPD to participate in a conversation, to sit on a sofa and be able to get up, or to eat with others while being unable to hold a knife and fork.

It's problematic when she visits us … she has … difficulty getting up from the sofa when she's with us and it makes the visits difficult, and then dragging her feet when she walks, not being able to lift her feet … it progressed quickly the last year … (Individual interview 14).

The care partners pointed out that they were the ones who had to be engaged and motivated to make social activities happen. The care partners lacked information on how to encourage social contacts for their partners and guidance on how to find social activities the PwPD could participate in and that could be adapted to the fluctuating symptoms of PD. They wished for advice on how to support their PwPD in social situations where the PD was obvious to others, might interfere with a social activity or might be potentially embarrassing.

I: I can see that the balance in the family has changed and shifted more and more to me and has ended up with me now, almost completely … He has been just as involved as I've been in discussions and talking, but now he's completely silent, and I can find that very difficult, because it has never been like that before.

II: We try to be out, but it requires someone to push him … now we're going to do this and now we're going to do that ….

III: Something that I can find stressful is that she has a very hard time and … she gets stuck in the floor when she has to walk, you're always afraid that she'll fall, and why hasn't anyone said that it can be like that? (Focus group interview 11).

### 3.3 To Find Reassurance Together With Others

To find reassurance together with others in similar situations, care partners described how they wanted information on where to meet other care partners. They expressed a need for discussion groups, either digital or in‐person, depending on their life circumstances. Sometimes they talked to other care partners living with family members with other diagnoses, but they found that other care partners had trouble understanding challenges and situations specific to PD.

I: Is this the first time you have met other carers?

II: Yes, you could say it's the first time, yes, and I have to say it's something I've missed.

III: Yes, I can say that I agree with you. It would have been nice … to have … talked to … you feel … even if you have a lot of people around you, you can feel alone in this, I think. The others do not understand what you're talking about (Focus group interview 11).

The care partners described feeling increasingly alone as the PD of their family member progressed. They expressed feeling a lack of someone who could listen and engage with their situation when the disease made it hard for their PwPD to talk and interact as before. When the symptoms of the PwPD increased and the care partners did not know who to contact, they felt vulnerable and abandoned, as they did not know how to best help the PwPD. Some care partners had a healthcare contact they could call, such as a PD nurse, neurologist or physiotherapist, while others did not know who to contact. They described needing information on who to contact when they needed to discuss medication, especially when changes in symptoms were very rapid.

I: What I miss very much is this contact … that there was a nurse that I felt I could call, who got involved or cared when I called … That I felt listened to, that I could discuss with someone. I can't discuss with him (the PwPD): ‘What's best for you?’ It can be 6 months; it can be 1 year between visits to the physician. That's probably what I miss most, that I would like to have a sounding board.

II: You need … help here and now, that someone can answer the question, ‘what should I do?’. It's security (Focus group interview 15).

Despite their family members having been diagnosed with a long‐term disease, the care partners said that they were rarely asked by healthcare professionals how they were managing or how they felt. They expressed a need for professionals to pay attention to their situation, to acknowledge that they needed support and help, and to invite them to participate as a resource and acknowledged partner in care appointments.

I have to say that, even though we've been in healthcare for so many years with Parkinson's disease, nobody has ever asked me as a carer … Yes, what it's like to be a carer of a person with Parkinson's and that maybe you could get some support and help (Focus group interview 15).

### 3.4 To Cope With Feelings of Uncertainty

To cope with feelings of uncertainty, care partners expressed a need for information about how to cope with the feelings, thoughts and worries that could arise when living close to a PwPD. They noted that, as PD progressed, feelings of uncertainty increased; they had more questions and thoughts about what the future might bring. The care partners explained that they understood PD to be a disease characterised by uncertainties that affect people in different ways. This uncertainty made it difficult for them to know what to expect, and they would have liked information about feelings of insecurity that could arise—that is, to be told it is normal to feel and think that it is uncertain to live with PD, and to learn how to cope with it.

There are a lot of thoughts … that you want to do as much as you can, when you know that it's like … it's not possible to cure, but you know… well … but you don't know the pace … so there's a lot of thinking about it [PD]. When is it time for this? Or when will this not work? How long will this work? People are affected in different ways … there are so many thoughts about it that would have been good to know more about (Individual interview 13).

The care partners described how everyday decisions were gradually placed on them. For example, they noticed that their PwPD were increasingly insecure and wanted them to make decisions for them. The care partners found it challenging to make such decisions because it was difficult to know what was best, especially regarding medication; decision‐making was experienced as an act of balance.

When you must do something, she always asks me, What do you think? Before, she never … she did it herself … it's very much now that I'm going say the last word and there's an uncertainty that comes. It's hard to make decisions for both her and me (Focus group interview 15).

The care partners described a need for information about how to cope with thoughts about planning for the future. The care partners expressed concerns about what would happen to the PwPD if they themselves became ill or if something happened that prevented them from providing help and support. They described how difficult it was to cope with feelings of uncertainty and fear when the progress of PD affects each person different.

I: I think a lot about if something happens to me … We have children … but I'm still afraid, yes, I feel that the future is uncertain … and if something happens to me, what will happen to him?

II: The future is uncertain, I think you summarise it well …

III: Yes, you'll have to look at the next few months, perhaps.

II: Yes. Even though the physician has said that … the progress is slow, because she's had it for many years … but still, it's a bit … well, I don't know, it doesn't feel … it doesn't feel safe for me … (Focus group interview 11).

## 4 Discussion

This study explored care partners' needs for information when caring for a family member with PD. The results revealed four themes that can be used as a guide when planning to provide information to care partners: (1) to be prepared when symptoms limit daily life, (2) to preserve participation and social life, (3) to find reassurance together with others and (4) to cope with feelings of uncertainty. The findings in our study align closely with Andershed and Ternestedt's [24] theoretical framework based on four studies in palliative care, particularly in terms of how care partners' experiences are shaped by whether their need for information and support is met. When care partners receive timely, relevant information—such as how to prepare for changes in everyday life, maintain social participation and cope with uncertainty—they are better equipped to remain centred, feeling supported and able to manage their caregiving role meaningfully.

A central finding in our study is the care partners' need for information throughout the trajectory of PD. The care partners called for repeated information to help them be aware of and manage the physical, emotional, social and existential aspects of PD that could affect themselves and the PwPD at different stages of the disease. This need for information when living with a long‐term illness has been described in previous studies. Social and physical support are often discussed in research in relation to care partners [25, 26], while emotional and existential support are less prominent [27]. As emotional and existential concerns increasingly emerge among care partners of family members living with a progressive disease, encompassing issues of meaning, loss, freedom, and end of life [15], it is essential that emotional and existential support is integrated into the care of PwPD and their care partners. The results of the present study call for a holistic approach in which the care partners' physical, social, emotional and existential needs are met. In person‐centred care, a holistic approach is central [28], and research calls for personalised care management for PwPD and their care partners as a dyadic support [29]. Therefore, a holistic approach has been suggested when developing supportive structures for the care partners of PwPD [30]. One study suggests that holistic support can be provided by creating what they call ‘a roadmap’ – that is, a shared plan for the future, together with the PwPD and the care partner [11]. Such an approach may enable care partners to feel prepared, involved and acknowledged over a prolonged period of time, which aligns with the results of this study. The framework by Andershed and Ternestedt [24] reveals the importance of ‘to know’ as a prerequisite for ‘to be’ and ‘to do’. Our findings show that care partners want personalised information directed at them in their specific circumstances together with the PwPD. Such support could be a prerequisite to *be* there for their PwPD and to *do* what is important—both for their own well‐being and for the PwPD.

Another central finding is the care partners' need for preparedness. To feel prepared for whatever PD may bring to their everyday life, the care partners endeavour to feel included and involved in the care of their PwPD. The care partners expressed that they wanted healthcare professionals to pay attention to their situation and to acknowledge that they might need to be invited to participate as a resource and partner in care appointments. This is in line with existing research showing that participation is a vital part of PD care, both for the PwPD and for the care partner [31, 32]. For example, a recent study showed that Parkinson's nurse specialists play a key role in supporting care partners and PwPD by empowering their ability to manage the challenges of PD [32]. In addition, the results of the current study advance the understanding of what the information needs to entail, why it is needed and how information could be delivered to meet the care partners' needs and preferences. A follow‐up of adherence to the national guidelines for PD care in Sweden shows that most PwPD receive appropriate medication, whereas access to regular follow‐up and support from different professionals within the care team was insufficient. Notably, support for care partners was not explicitly addressed in the follow‐up [22].

The framework by Andershed and Ternestedt [24] shows that involvement and participation are vital in feeling recognised and secure as a care partner. A central aspect of participation is shared decision‐making. In a person‐centred approach, the concepts of ‘shared decision‐making’ and ‘shared understanding’ are used to refer to mutuality and participation in an encounter [28]. However, a meta‐synthesis on shared decision‐making showed that it is not a ‘simple’ thing to experience meetings as ‘shared’ in a healthcare context [33]. Knowledge is often mentioned as important in the meeting between professionals and patients, but the meta‐synthesis showed that it is not enough to feel involved. For an encounter to truly be participated in, power relations must be equalised by the different participants having similar perceptions of the situation and access to the same information [33]. In the current study, the results reveal how the care partners live in tension between striving to be prepared to deal with different symptoms that might appear in the PwPD and searching for answers without a clear communication pathway. However, they do not know when or even if different symptoms will affect their PwPD and care partners expressed that they did not know who to contact for advice and guidance. Their circumstances can be metaphorically described as ‘fumbling in the dark’ [24]. Instead, to enable care partners to be ‘in the light’, continuous information throughout the trajectory of PD is central. This information must be based on a holistic approach and on shared decision‐making between healthcare professionals, the PwPD and the care partner. According to WHO [1] this is not a solely challenge in one specific country but rather a need for PwPD and care partners in many countries.

### 4.1 Methodological Considerations

To address trustworthiness [34], a possible threat to the dependability is that at the time of consent, care partners were only asked about the duration of PD although further questions regarding disease stage could have been posed. This was deliberately limited as the interviews focused on care partners rather than the PwPD. The decision was guided by ethical and person‐centred considerations, with the researchers remaining attentive to the care partners' needs and preferences during data collection. A further potential threat to the dependability is that the interviews were conducted in three different formats: in a group, dyadic, or individually. However, these different formats made it possible for all the care partners who wished and felt like they had important information on the topic to share to participate, even if their PwPD was in a late stage of PD and they were reluctant to leave the home, or they had work to do. In addition, the same interview guide was used in all interviews, and the same researchers conducted all interviews and analysed the data, first individually and then together. However, various formats of interviews provided different data for analysis. For example, group interviews provided more discussions and interaction between participants. Therefore, the analysis started by reading the group interviews, followed by the dyadic and the individual. To verify credibility, the results are presented with a description of the analysis, a table, and quotes from the interview transcripts. Concerning confirmability, the two researchers conducting the interviews are registered nurses, with one being specialised in PD. Therefore, it was important to involve two more researchers to prevent preunderstandings from colouring the analysis. In addition, three of the researchers have extensive experiences of conducting qualitative research. Throughout the study, the researchers engaged in reflexive discussions regarding their professional backgrounds and preunderstandings, particularly in relation to data collection and interpretation. While such preunderstandings may pose a potential risk of bias, they can also be regarded as a strength. The researchers' clinical and methodological expertise enabled sensitivity to flexibility in adapting different forms of interviews and attentiveness to the need for follow‐up questions during the interviews. A limitation to the transferability of this work is that the study only was conducted in a Swedish healthcare context and that it is an initial study. Still, a table with the demographics of the participants and a description of the setting is provided to enable the reader to determine whether the results are transferable. More research is needed, such as intervention studies, regarding the provision of information to care partners.

## 5 Conclusions

This study highlights a need for personalised information tailored to the care partners of PwPD. The findings identify four themes representing care partners' information needs: preparedness, social participation, emotional support, and coping with uncertainty. The information does not only need to convey facts, but also create security, enable participation, confirm existential and emotional experiences, and support meaningful care over a prolonged time. Information that supports ‘knowing’ in order to enable ‘being’ and ‘doing’, in line with Andershed and Ternestedt's framework [24]. The results of the study can be seen as an inspiration for the co‐creation of material for care partners that can be used in clinical work and in individual care meetings, as well as being available online and thus accessible to more PwPD and their care partners. That is, the results underscore the importance of holistic, information‐based interventions developed through collaboration between the team of healthcare professionals, the PwPD, and the care partner. The implications for practice suggest that no single intervention will meet the needs of all care partners. Instead, flexible and adaptable approaches are required, taking into account care partners' individual circumstances and everyday contexts. However, both the content of the information and the modes of delivery should be co‐developed with care partners. The four identified themes may serve as a conceptual framework for the development of future information‐based interventions.

## Author Contributions

H.L., C.H. and M.H. designed the study. H.L. and C.H. conducted the interviews. H.L., C.H. and M.H. analysed the material. H.L. wrote the first draft, and C.H., M.H. and P.H. contributed substantially to its revision. All authors participated in reviewing and rewriting the manuscript and approved the final version for publication.

## Funding

This study was supported by the Research Platform for Collaboration for Health, Kristianstad University.

## Conflicts of Interest

The authors declare no conflicts of interest.

## Supporting information


**Data S1:** COREQ (COnsolidated criteria for REporting Qualitative research) Checklist.

## Data Availability

The data generated and analysed during this study are in Swedish; while not publicly available, they are available from the corresponding author upon reasonable request.
